# Development and validation of prediction model for fall accidents among chronic kidney disease in the community

**DOI:** 10.3389/fpubh.2024.1381754

**Published:** 2024-05-30

**Authors:** Pinli Lin, Guang Lin, Biyu Wan, Jintao Zhong, Mengya Wang, Fang Tang, Lingzhen Wang, Yuling Ye, Lu Peng, Xusheng Liu, Lili Deng

**Affiliations:** ^1^The Second Clinical College of Guangzhou University of Chinese Medicine, Guangzhou, China; ^2^The Fourth Clinical College of Guangzhou University of Chinese Medicine, Guangzhou, China; ^3^School of Nursing Hunan University of Chinese Medicine, Changsha, China; ^4^Department of Chronic Disease Management, The Second Affiliated Hospital of Guangzhou University of Chinese Medicine (Guangdong Provincial Hospital of Traditional Chinese Medicine), Guangzhou, China; ^5^Department of Nephrology, The Second Affiliated Hospital of Guangzhou University of Chinese Medicine (Guangdong Provincial Hospital of Traditional Chinese Medicine), Guangzhou, China; ^6^School of Nursing, Guangzhou University of Chinese Medicine, Guangzhou, China

**Keywords:** falls, chronic kidney disease, CHARLS, predictive model, nomogram

## Abstract

**Background:**

The population with chronic kidney disease (CKD) has significantly heightened risk of fall accidents. The aim of this study was to develop a validated risk prediction model for fall accidents among CKD in the community.

**Methods:**

Participants with CKD from the China Health and Retirement Longitudinal Study (CHARLS) were included. The study cohort underwent a random split into a training set and a validation set at a ratio of 70 to 30%. Logistic regression and LASSO regression analyses were applied to screen variables for optimal predictors in the model. A predictive model was then constructed and visually represented in a nomogram. Subsequently, the predictive performance was assessed through ROC curves, calibration curves, and decision curve analysis.

**Result:**

A total of 911 participants were included, and the prevalence of fall accidents was 30.0% (242/911). Fall down experience, BMI, mobility, dominant handgrip, and depression were chosen as predictor factors to formulate the predictive model, visually represented in a nomogram. The AUC value of the predictive model was 0.724 (95% CI 0.679–0.769). Calibration curves and DCA indicated that the model exhibited good predictive performance.

**Conclusion:**

In this study, we constructed a predictive model to assess the risk of falls among individuals with CKD in the community, demonstrating good predictive capability.

## 1 Introduction

Chronic kidney disease (CKD) has an estimated global prevalence of approximately 9.1% among adults (1). The prevalence of CKD rises linearly with age, with individuals over 60 accounting for more than 60% of all cases (2). CKD often results in systemic complications, causing decreased physical strength, cognitive decline, osteoporosis, muscle atrophy, and other issues that elevate the risk of falls (3–5). Studies have demonstrated that individuals with CKD face an elevated risk of falling compared to those without the condition, indicating an elevated risk of falls among individuals with chronic kidney disease (6–9).

Falls resulting in injuries may lead to fatalities and long-term disabilities, adversely affecting the quality of life for those impacted (10). Epidemiological studies confirm that falls are universally recognized as the second most common cause of unintentional injury-related fatalities (11). Individuals with CKD often experience conditions such as renal osteodystrophy, leading to compromised bone health. This renders CKD patients more susceptible to fractures in the event of a fall compared to the general population (4, 12, 13). Furthermore, even non-injurious falls may instill fear of falling in patients, increasing the risk of recurrent falls (14, 15). These underscore the critical importance of early monitoring and fall prevention in individuals with CKD.

The treatment of CKD is a long-term process, and patients typically reside within the community, making it challenging for healthcare professionals to monitor and manage their conditions in real-time. Therefore, the development of a reliable model within the community could identify CKD individuals at a heightened risk of falls, enabling personalized management strategies.

The occurrence of falls in individuals with CKD is a result of the synergistic impact of various factors. Previous studies have indicated that weaknesses, muscle wasting, depression, cognitive decline, electrolyte imbalances, anemia, and other symptoms commonly associated with CKD are related to the occurrence of falls (5, 16–18). In this study, the objective is to develop and validate models for predicting the occurrence of falls among individuals with CKD in the community. The aim is to facilitate the early identification of fall risks and enhance alertness to their potential occurrence.

## 2 Methods

### 2.1 Study population and design

The data for this study was sourced from the China Health and Retirement Longitudinal Study (CHARLS) (19). which is a comprehensive nationwide survey conducted by the National Institute of Development of Peking University. CHARLS is designed to collect demographic and health-related data from individuals aged 45 and above across 28 provinces in China. This study was carried out in compliance with ethical standards and received approval from the Biomedical Ethics Review Committee of Peking University. All participants provided written informed consent, and the study was assigned the ethical approval number IRB00001052-11015.

**Figure 1:**
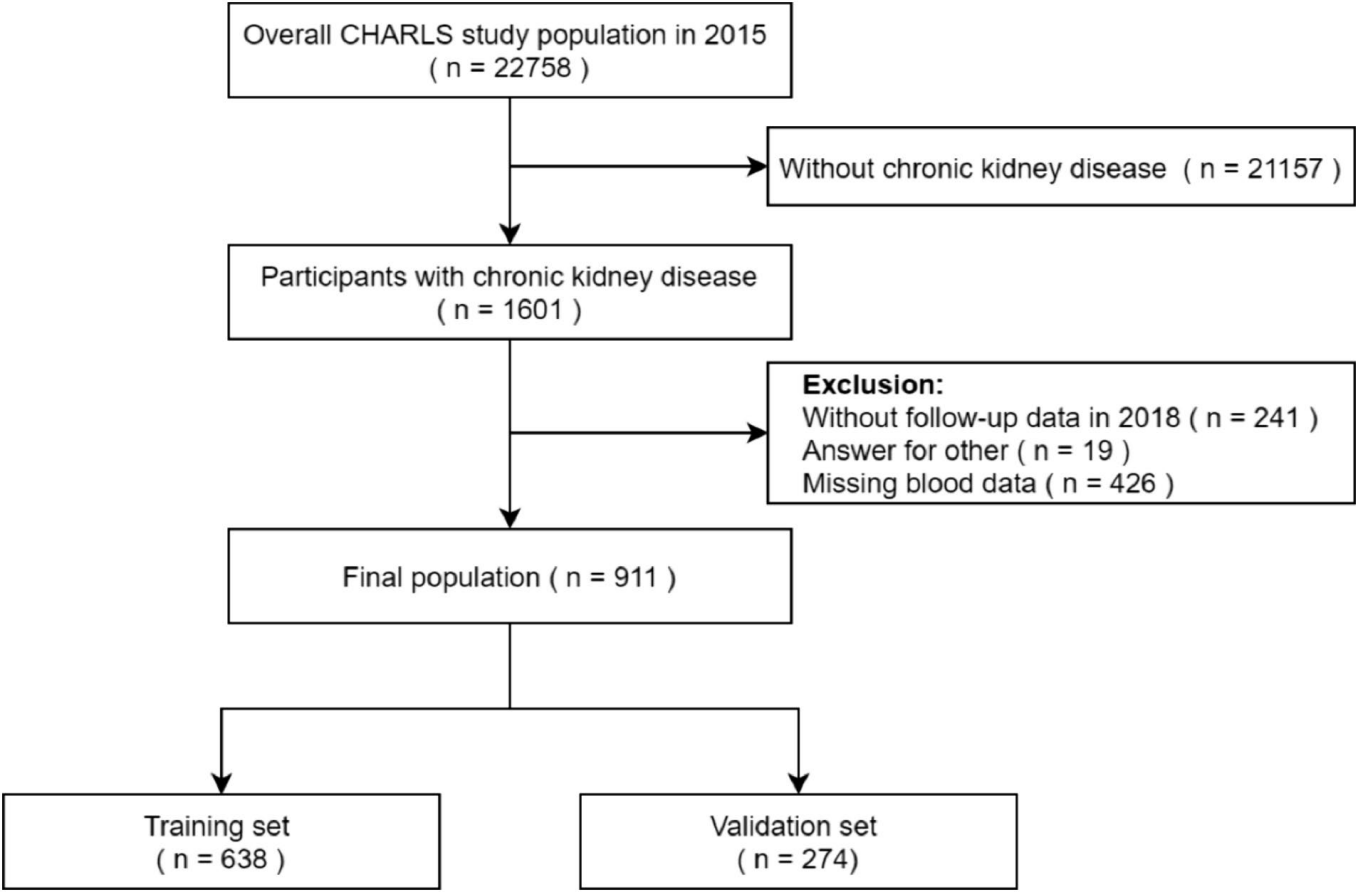
Flowchart of participants selection.

For the present analysis, we included participants who self-reported chronic kidney disease in 2015. We excluded individuals who lacked information on falls during the 2017–2018 survey. Additionally, participants with missing blood data and answered by others were also excluded. Final study cohort comprising 911 participants (Figure 1).

### 2.2 Fall accidents

Participants reported these incidents through a specific question in the 2017–2018 survey: “Have you experienced any falls since your last visit?” Participants provided responses, indicating either “yes” or “no.”

### 2.3 Variables

The variables included in the analysis encompassed a wide range of factors. These factors covered demographic information such as age, gender, marital status, disability and body mass index (BMI). Behavioral data was considered, including alcohol consumption status, smoking status, mobility, toilet seat usage, fall down experiences, dominant handgrip strength, pain status, night sleep duration, as well as activities of daily living (ADL) and instrumental activities of daily living (IADL).

Health status was thoroughly assessed, taking into account blood pressure, CESD-10 score, cognitive score, and various comorbidities such as hypertension, dyslipidemia, diabetes, cancer, heart disease, stroke, depression, memory-related diseases, arthritis, asthma, and glaucoma. In addition, several blood indicators were included, such as white blood cell count (WBC), hemoglobin, platelet count, triglycerides (TG), blood urea nitrogen (BUN), high-density lipoprotein (HDL), low-density lipoprotein (LDL), glucose, uric acid (UA), cystatin C, C-reactive protein, and hemoglobin A1c (HbA1c). The estimated glomerular filtration rate (eGFR) was also considered in the analysis.

Disability encompasses physical disabilities, brain damage/mental retardation, or vision problems. BMI was calculated using the standard formula. Participants with a BMI > 45 were considered outliers and were excluded from further analysis. Mobility included walking sticks, travel devices, manual wheelchairs, and electric wheelchairs. The dominant handgrip strength was measured using a hydraulic handgrip dynamometer, specifically the Yuejian TM WL-1000 dynamometer. To determine the dominant hand, participants were asked the question, “Which is your dominant hand?” The handgrip strength was then recorded by averaging the results of two separate measurements. Participants with handgrip strength 0 kg were identified as outliers and excluded from further analysis. The cognitive score in CHARLS was determined through telephone interviews assessing cognitive status (TICS-10), a visuospatial ability test, and an episodic memory capacity test. A lower score indicates a higher risk of cognitive impairment. The CESD-10 score was utilized to evaluate depression. The total cumulative score on this scale is 30, with a score of 10 or higher indicating a high risk of depression. eGFR calculated was based on the eGFR. MDRD. Chinese (20).

### 2.4 Statistical analysis

All statistical analyses were conducted using the R statistical software package, version 4.3.1 (http://www.R-project.org, The R Foundation), and Free Statistics software, version 1.9.1. Continuous variables, including normally distributed data presented as means ± standard deviations and skewed data expressed as medians with interquartile ranges, were analyzed in this study. Categorical variables are reported as percentages. Baseline characteristics were compared using the *t*-test for normally distributed continuous variables and the rank sum test for skewed continuous variables. The chi-square test or Fisher’s exact test was employed for the analysis of categorical variables.

Multiple interpolation was used to handle missing data. Data were randomly divided into training (*n* = 638) and validation (*n* = 273) sets, according to a ratio of 7:3. Training set data were analyzed by least absolute shrinkage and selection operator (LASSO) regression and logistic regression analysis to select predictors of fall accidents in people with CKD.

In this study, the area under the receiver operating characteristic (ROC) curve (AUC) was used to determine the discrimination ability of the model. Calibration curves were used to determine the degree of agreement between predicted probabilities and observed outcomes. Decision curve analysis (DCA) was used to assess clinical validity. A nomogram was used to illustrate the risk of fall in individuals with CKD. Internal consistency of the discrimination and calibration performance measures was evaluated by 10-fold cross-validation. All statistical tests were two-tailed, and a significance level of *p* < 0.05 was considered statistically significant.

## 3 Results

### 3.1 Baseline characteristics

Our study comprised 911 participants with CKD. The mean age was 61.2 ± 9.0 years, 464 (50.9%) were male. The incidence of fall accidents among participants with CKD was 30.0%. The baseline characteristics were summarized in Supplementary Table S1. Maximum missing values for all variables extracted did not exceed 6.5%. To address this, we applied data multiple interpolation techniques. After divide into training (*n* = 638) and validation (*n* = 273) sets, we obtained baseline characteristics of participants were showed in Table 1.

**Table 1:** Baseline characteristics of participants.

Variables	Training set			Validation set		
	Non-fall (*n* = 463)	Fall (*n* = 175)	*p*	Non-fall (*n* = 206)	Fall (*n* = 67)	*p*
Gender, *n* (%)	244 (52.7)	82 (46.9)	0.188	108 (52.4)	30 (44.8)	0.277
Age, Mean ± SD	60.7 ± 8.7	62.6 ± 8.8	0.014	60.8 ± 9.1	61.4 ± 11.3	0.709
Married Status. *n* (%)	418 (90.3)	144 (82.3)	0.005	189 (91.7)	58 (86.6)	0.21
Disability, *n* (%)	148 (32)	60 (34.3)	0.577	60 (29.1)	31 (46.3)	0.01
Alcohol Status, *n* (%)	176 (38)	68 (38.9)	0.845	61 (29.6)	21 (31.3)	0.788
Smoke Status, *n* (%)	176 (38)	71 (40.6)	0.554	76 (36.9)	19 (28.4)	0.203
BMI, *n* (%)			0.222			0.462
<18.5	24 (5.2)	16 (9.1)		12 (5.8)	2 (3)	
18.5–24.9	262 (56.6)	100 (57.1)		107 (51.9)	42 (62.7)	
25–29.9	149 (32.2)	52 (29.7)		72 (35)	18 (26.9)	
≥30	28 (6)	7 (4)		15 (7.3)	5 (7.5)	
Mobility, *n* (%)	19 (4.1)	19 (10.9)	0.001	6 (2.9)	12 (17.9)	<0.001
Toilet Seat Usage, *n* (%)	11 (2.4)	7 (4)	0.287	7 (3.4)	3 (4.5)	0.711
Fall Down Experience, *n* (%)	94 (20.3)	89 (50.9)	<0.001	36 (17.5)	33 (49.3)	<0.001
Glaucoma, *n* (%)	3 (0.6)	3 (1.7)	0.354	2 (1)	1 (1.5)	0.572
Pain, *n* (%)	206 (44.5)	98 (56)	0.009	90 (43.7)	45 (67.2)	<0.001
Night sleep duration, Mean ± SD	6.0 ± 2.0	5.6 ± 2.1	0.011	6.3 ± 1.9	5.7 ± 2.3	0.025
ADL, *n* (%)			<0.001			0.045
Independent	338 (73)	94 (53.7)		147 (71.4)	39 (58.2)	
Dependent	125 (27)	81 (46.3)		59 (28.6)	28 (41.8)	
IADL, *n* (%)			<0.001			0.127
Independent	331 (71.5)	93 (53.1)		138 (67)	38 (56.7)	
Dependent	132 (28.5)	82 (46.9)		68 (33)	29 (43.3)	
CESD-10 score, Mean ± SD	20.5 ± 5.9	22.4 ± 6.0	<0.001	19.8 ± 5.8	23.7 ± 6.5	<0.001
Cognitive score, Mean ± SD	10.9 ± 3.9	9.9 ± 4.2	0.007	11.0 ± 3.8	10.1 ± 4.0	0.094
Waist, Mean ± SD	84.7 ± 15.8	83.8 ± 15.5	0.51	87.1 ± 12.6	84.6 ± 17.5	0.21
Dominant handgrip (kg), Mean ± SD	29.8 ± 9.5	26.1 ± 9.6	<0.001	29.3 ± 9.5	25.9 ± 8.4	0.009
eGFR, Mean ± SD	99.8 ± 28.5	100.0 ± 26.5	0.93	99.6 ± 28.2	104.0 ± 26.2	0.253
SBP, Mean ± SD	127.1 ± 18.3	128.3 ± 21.2	0.5	127.7 ± 19.6	130.2 ± 20.1	0.381
DBP, Mean ± SD	75.5 ± 11.3	74.8 ± 12.1	0.504	75.8 ± 11.3	76.9 ± 12.5	0.489
Pulse, Mean ± SD	73.1 ± 10.7	72.5 ± 10.5	0.489	75.0 ± 10.2	74.7 ± 10.3	0.821
White blood cell, Mean ± SD	6.0 ± 1.8	5.7 ± 1.6	0.072	6.3 ± 2.0	6.3 ± 2.9	0.791
Hemoglobin, Mean ± SD	13.8 ± 2.1	13.5 ± 1.8	0.115	13.9 ± 2.2	13.6 ± 2.0	0.356
Platelets, Median (IQR)	191.0 (154.0, 237.5)	183.0 (145.5, 229.5)	0.079	205.5 (162.2, 251.8)	194.0 (164.5, 240.5)	0.22
TG, Median (IQR)	110.6 (81.0, 166.8)	110.6 (78.8, 160.2)	0.365	116.4 (80.8, 163.7)	99.1 (85.8, 162.8)	0.735
BUM, Median (IQR)	15.4 (12.8, 18.5)	15.4 (13.2, 18.2)	0.515	14.8 (12.1, 18.2)	14.8 (12.6, 17.2)	0.969
HDL-C, Median (IQR)	50.2 (42.7, 58.7)	52.1 (44.0, 60.2)	0.176	49.2 (43.7, 56.4)	53.3 (42.9, 59.7)	0.231
LDL-C, Median (IQR)	100.4 (81.1, 117.8)	102.7 (84.6, 119.1)	0.377	100.4 (85.3, 118.9)	103.5 (88.6, 127.8)	0.236
Glucose, Median (IQR)	95.5 (88.3, 104.5)	95.5 (88.3, 104.5)	0.523	95.5 (88.3, 102.7)	93.7 (85.6, 99.1)	0.38
UA, Median (IQR)	4.9 (4.0, 5.9)	4.7 (4.1, 5.8)	0.762	4.8 (4.0, 5.9)	4.9 (4.0, 6.0)	0.955
Cystatin C, Median (IQR)	0.8 (0.7, 1.0)	0.9 (0.7, 1.0)	0.474	0.8 (0.7, 1.0)	0.9 (0.8, 1.0)	0.564
C-reactive protein, Median (IQR)	1.4 (0.8, 2.6)	1.3 (0.7, 2.6)	0.627	1.4 (0.8, 2.8)	1.7 (0.8, 4.0)	0.339
HbA1c, Median (IQR)	5.8 (5.5, 6.1)	5.8 (5.5, 6.2)	0.175	5.8 (5.5, 6.2)	5.8 (5.5, 6.1)	0.708
Hypertension, *n* (%)	181 (39.1)	71 (40.6)	0.733	77 (37.4)	32 (47.8)	0.132
Dyslipidemia, *n* (%)	106 (22.9)	40 (22.9)	0.992	43 (20.9)	16 (23.9)	0.603
Diabetes, *n* (%)	65 (14)	30 (17.1)	0.326	26 (12.6)	7 (10.4)	0.635
Cancer, *n* (%)	11 (2.4)	3 (1.7)	0.768	8 (3.9)	4 (6)	0.496
Heart attack, *n* (%)	143 (30.9)	56 (32)	0.786	54 (26.2)	26 (38.8)	0.049
Stroke, *n* (%)	16 (3.5)	8 (4.6)	0.509	8 (3.9)	5 (7.5)	0.318
Depression disease, *n* (%)	15 (3.2)	3 (1.7)	0.424	5 (2.4)	3 (4.5)	0.41
Memory related disease, *n* (%)	18 (3.9)	13 (7.4)	0.063	5 (2.4)	5 (7.5)	0.069
Arthritis, *n* (%)	256 (55.3)	119 (68)	0.004	116 (56.3)	46 (68.7)	0.074
Asthma, *n* (%)	40 (8.6)	19 (10.9)	0.388	18 (8.7)	11 (16.4)	0.076

BMI, body mass index; ADL, activity of daily living; IADL, instrumental activities of daily living; CESD-10, Center for Epidemiologic Studies Depression Scale – 10 item version; SBP, systolic blood pressure; DBP, diastolic blood pressure; TG, triglycerides; BUM, blood urea nitrogen; HDL-C, high-density lipoprotein cholesterol; LDL-C, low-density lipoprotein cholesterol; UA, uric acid.

### 3.2 Prevalence of fall accidents and related risk factors

The prevalence of fall accidents was 26.6% (242/911). Univariate logistic regression analysis indicated that several factors, including age, marital status, mobility, fall down experience, pain status, nighttime sleep duration, ADL, IADL, CESD-10 score, cognitive score, dominant handgrip, and arthritis, differed significantly (*p* < 0.01) between participants with and without fall accidents. Further multivariable analysis demonstrated that fall down experience, CESD-10 score, dominant handgrip, and mobility were independent risk factors for fall accidents among CKD participants (Table 2).

**Table 2:** Logistic regression of fall accidents among chronic kidney disease.

Variable	Univariate		Multivariable	
	OR (95% CI)	*p*	OR (95% CI)	*p*
Gender	1.29 (0.96 ~ 1.73)	0.092	0.92 (0.53 ~ 1.62)	0.777
Age	1.02 (1 ~ 1.04)	0.025	0.99 (0.97 ~ 1.02)	0.656
Married status	1.94 (1.26 ~ 2.97)	0.002	1.55 (0.93 ~ 2.58)	0.092
Disability	1.34 (0.98 ~ 1.82)	0.065	0.83 (0.57 ~ 1.2)	0.317
Alcohol status	1.06 (0.78 ~ 1.44)	0.707	1.33 (0.9 ~ 1.95)	0.151
Smoke status	0.98 (0.72 ~ 1.33)	0.895	1.11 (0.73 ~ 1.69)	0.634
BMI				
18.5–24.9	ref			
<18.5	1.3 (0.71 ~ 2.36)	0.391	1.04 (0.52 ~ 2.11)	0.909
25–29.9	0.82 (0.59 ~ 1.15)	0.249	0.71 (0.46 ~ 1.09)	0.119
≥30	0.73 (0.37 ~ 1.42)	0.346	0.68 (0.3 ~ 1.55)	0.36
Mobility	3.78 (2.19 ~ 6.56)	<0.001	2.91 (1.45 ~ 5.81)	0.003
Toilet seat usage	1.56 (0.71 ~ 3.43)	0.269	0.54 (0.2 ~ 1.42)	0.208
Fall down experience	4.22 (3.07 ~ 5.78)	<0.001	3.5 (2.46 ~ 4.98)	<0.001
Glaucoma	2.23 (0.59 ~ 8.38)	0.234	1.87 (0.38 ~ 9.23)	0.441
Pain	1.82 (1.35 ~ 2.45)	<0.001	0.94 (0.63 ~ 1.38)	0.739
Night sleep duration	0.88 (0.82 ~ 0.95)	0.001	0.97 (0.89 ~ 1.06)	0.493
ADL				
Dependent	Ref.		Ref.	
Independent	2.16 (1.59 ~ 2.93)	<0.001	1.25 (0.83 ~ 1.89)	0.291
IADL				
Dependent	Ref.		Ref.	
Independent	1.99 (1.47 ~ 2.69)	<0.001	0.98 (0.64 ~ 1.49)	0.919
CESD-10 score	1.07 (1.04 ~ 1.1)	<0.001	1.04 (1.01 ~ 1.08)	0.012
Cognitive score	0.94 (0.91 ~ 0.98)	0.002	0.99 (0.95 ~ 1.04)	0.707
Waist	0.99 (0.98 ~ 1)	0.215	1 (0.98 ~ 1.01)	0.873
Dominant handgrip (kg)	0.96 (0.94 ~ 0.98)	<0.001	0.97 (0.94 ~ 0.99)	0.01
eGFR	1 (1 ~ 1.01)	0.502	1 (1 ~ 1.01)	0.27
SBP	1 (1 ~ 1.01)	0.305	1 (0.99 ~ 1.01)	0.919
DBP	1 (0.99 ~ 1.01)	0.827	1 (0.98 ~ 1.03)	0.69
Pulse	0.99 (0.98 ~ 1.01)	0.432	0.99 (0.97 ~ 1)	0.105
White blood cell	0.93 (0.86 ~ 1.01)	0.102	0.96 (0.87 ~ 1.06)	0.39
Hemoglobin	0.93 (0.87 ~ 1)	0.067	0.95 (0.86 ~ 1.05)	0.293
Platelets	1 (1 ~ 1)	0.1	1 (1 ~ 1)	0.065
TG	1 (1 ~ 1)	0.78	1 (1 ~ 1)	0.666
BUM	1 (0.97 ~ 1.03)	0.91	0.98 (0.95 ~ 1.02)	0.425
HDL	1.01 (1 ~ 1.02)	0.071	1.01 (0.99 ~ 1.02)	0.481
LDL	1 (1 ~ 1.01)	0.044	1.01 (1 ~ 1.01)	0.094
Glucose	1 (1 ~ 1.01)	0.636	1 (0.99 ~ 1)	0.278
UA	0.98 (0.89 ~ 1.08)	0.697	1.12 (0.97 ~ 1.3)	0.132
Cystatin C	1.02 (0.67 ~ 1.53)	0.937	1.06 (0.5 ~ 2.22)	0.884
C-reactive protein	1.01 (0.99 ~ 1.03)	0.358	1.02 (0.99 ~ 1.04)	0.133
HbA1c	1.12 (0.99 ~ 1.27)	0.063	1.21 (0.99 ~ 1.47)	0.066
Hypertension	1.18 (0.88 ~ 1.59)	0.276	1.02 (0.68 ~ 1.53)	0.922
Dyslipidemia	1.05 (0.74 ~ 1.49)	0.782	1.07 (0.68 ~ 1.68)	0.76
Diabetes	1.15 (0.76 ~ 1.73)	0.518	0.97 (0.57 ~ 1.67)	0.915
Cancer	1.02 (0.42 ~ 2.46)	0.966	0.63 (0.22 ~ 1.85)	0.403
Heart attack	1.23 (0.9 ~ 1.68)	0.2	1.01 (0.68 ~ 1.49)	0.979
Stroke	1.53 (0.76 ~ 3.05)	0.231	1.31 (0.57 ~ 3)	0.519
Emotional	0.82 (0.33 ~ 2.08)	0.683	0.27 (0.09 ~ 0.82)	0.021
Memory related disease	2.26 (1.2 ~ 4.26)	0.012	1.63 (0.75 ~ 3.54)	0.214
Arthritis	1.71 (1.25 ~ 2.33)	0.001	1.16 (0.81 ~ 1.66)	0.415
Asthma	1.49 (0.93 ~ 2.38)	0.094	1.08 (0.61 ~ 1.91)	0.797

BMI, body mass index; ADL, activity of daily living; IADL, instrumental activities of daily living; CESD-10, Center for Epidemiologic Studies Depression Scale – 10 item version; SBP, systolic blood pressure; DBP, diastolic blood pressure; TG, triglycerides; BUM, blood urea nitrogen; HDL-C, high-density lipoprotein cholesterol; LDL-C, low-density lipoprotein cholesterol; UA, uric acid.

### 3.3 Predictive model development

**Figure 2:**
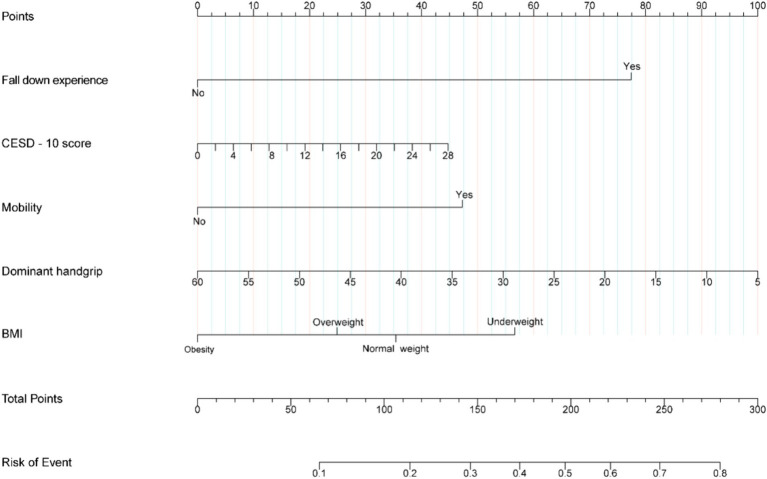
Nomogram of prediction model for fall accidents among chronic kidney disease.

To eliminate potential confounders and simplify the model, we employed LASSO regression analysis to identify the best predictors of the model. The variables considered in this model included fall down experience, ADL, IADL, mobility, dominant grip, marital status, and CESD-10 score as predictive factors (Supplementary Figure S1). This predictive model is intended for application in community populations, and for enhanced user-friendliness, we selected fall down experience, BMI, mobility, dominant handgrip, and depression as predictors in the model development process. Finally, the predictive model was developed and presented using a nomogram (Figure 2).

**Figure 3:**
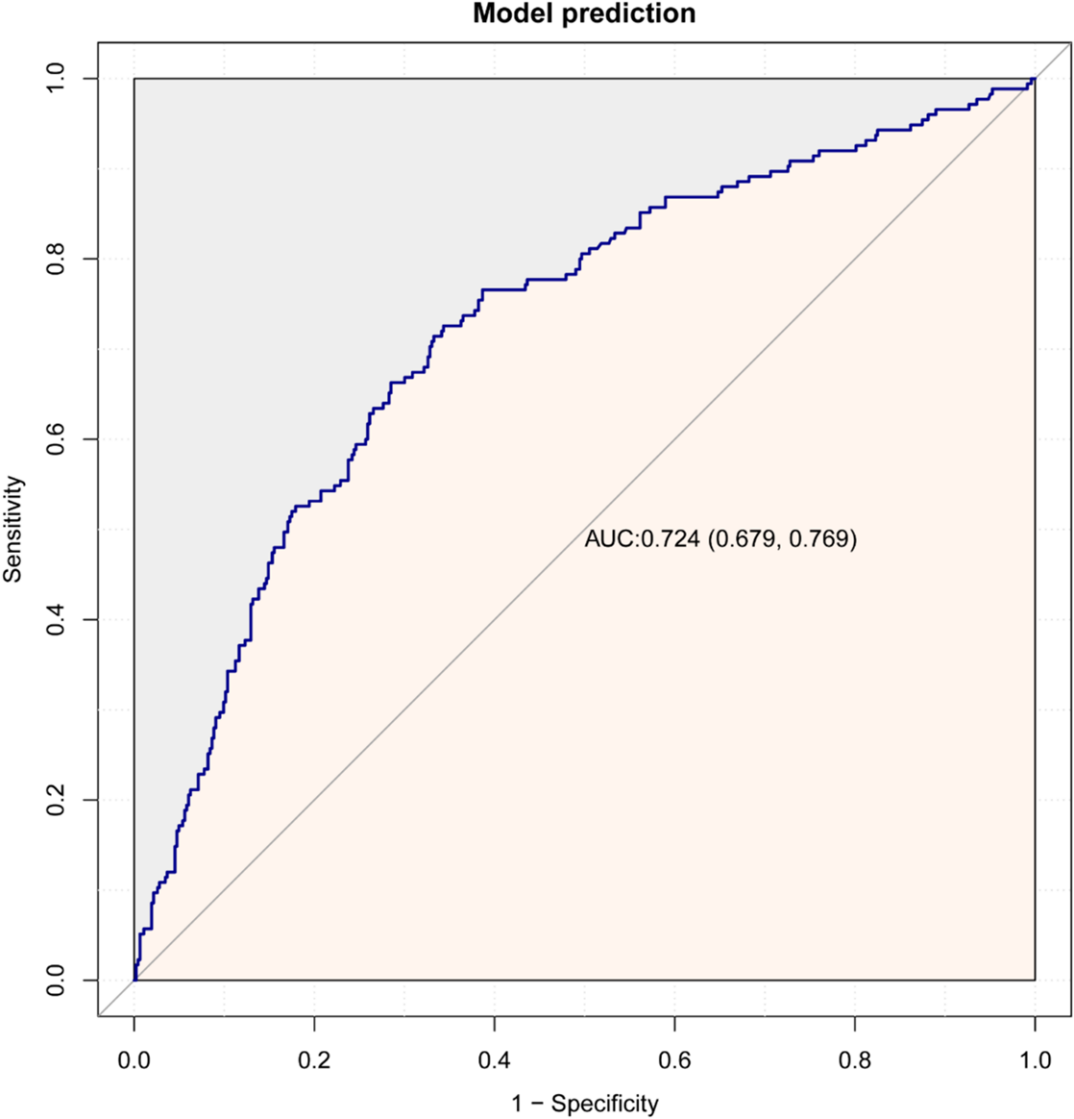
ROC curves plot for the training dataset.

**Figure 4:**
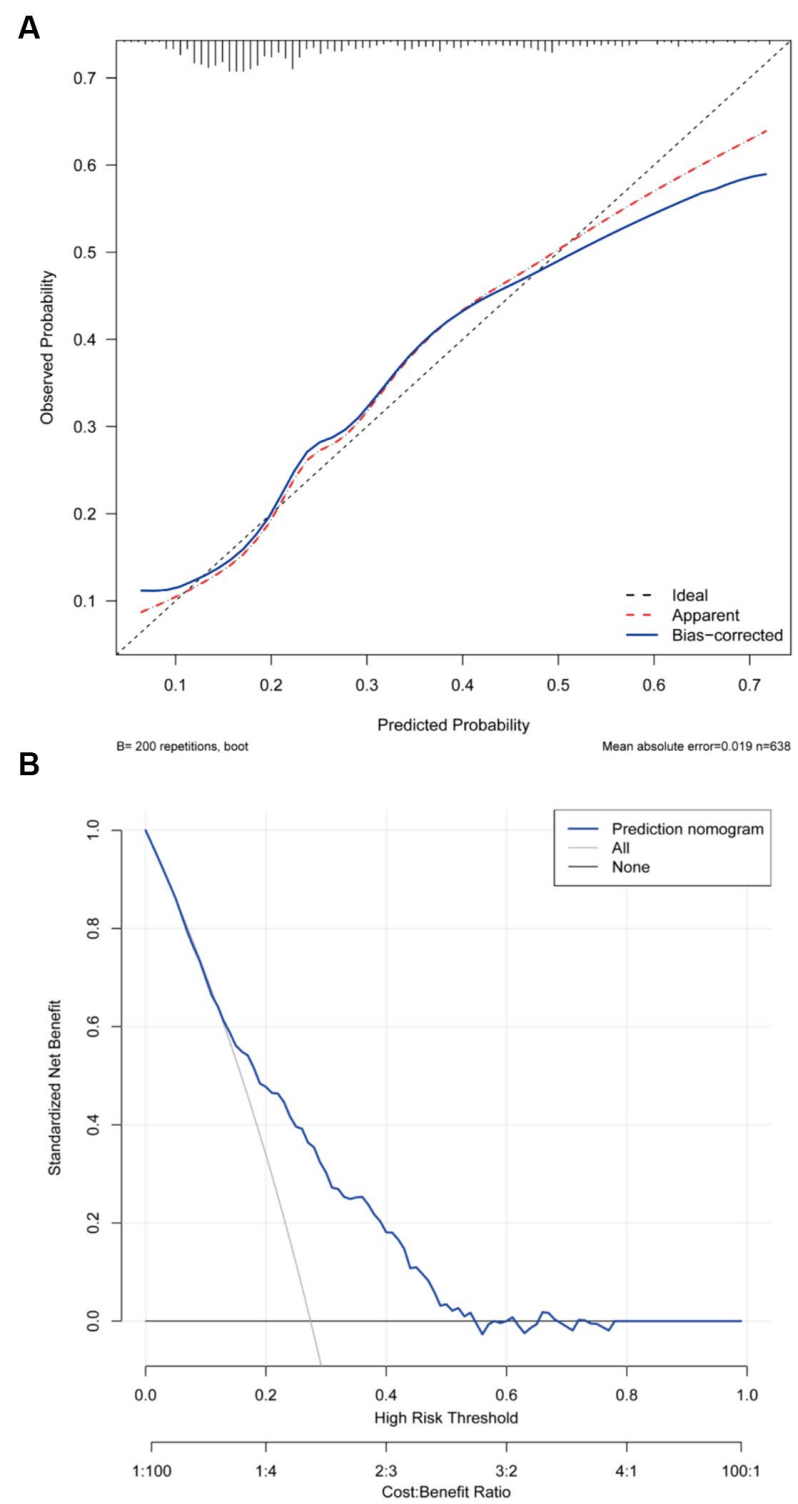
**(A)** Calibration plot for the training dataset. **(B)** Decision curve plot for the training dataset.

As shown in Figure 3, the predictive model achieved an AUC value of 0.724 (95% CI = 0.679–0.769), and the calibration curve illustrated that the model exhibited a good fit (*χ^2^* = 5.293, df = 7, *p* = 0.726). The clinical validity of the model was evaluated using the DCA method, and the results showed that the training set exceeded the extreme cases, indicating that the nomogram model provided superior net benefit and predictive accuracy (Figure 4).

### 3.4 Predictive model validation

Internal validation using a validation set demonstrated that the discrimination AUC, calibration *χ^2^*, and calibration slope means were similar to those obtained from the models for the entire population. This indicates good internal consistency for our equations. The DCA curve demonstrated the model’s clinical validity (Supplementary Figures S2–S4). Furthermore, we conducted an internal validation using a 10-fold cross-validation, and the results remained stable (the mean AUC = 0.719).

## 4 Discussion

In this study, fall down experience, BMI, mobility, dominant handgrip, and depression were selected as predictor factors to develop the predictive model for fall accidents among individuals with CKD in the community. The model demonstrated excellent performance in predicting fall risk, exhibiting robust internal consistency and external validation. To our knowledge, no other predictive model for fall accidents has been developed and validated among individuals with CKD in the community.

The occurrence of falls in patients with CKD is a consequence of the combined effects of multiple factors. This study revealed that a history of falling, BMI, mobility, dominant handgrip, and depression were predictors of fall accidents in individuals with CKD.

Having experienced a fall previously significantly increases the risk of subsequent fall accidents. This finding aligns with conclusions drawn from several studies involving the elders (21, 22). Currently widely applied fall risk prediction models for hospitalized older adult individuals similarly underscore the potential risk associated with a history of falling in predicting fall accidents. Studies on fall incidents among patients with liver cirrhosis and diabetes similarly utilize fall history as a predictive factor, highlighting the independent correlation between fall history and the occurrence of falls (23, 24). This correlation remains significant among individuals with CKD. It can be attributed to the fact that those with CKD, who have a history of falls, may be more prone to developing a fear of falling, which leads to reduced physical activity. The reduced physical activity contributes to diminished muscle function, thereby increasing the risk of falls (14). The use of mobility aids follows a similar pattern; their use is often considered in fall risk assessments, and it has been observed that the use of mobility aids decreases activity levels in individuals with CKD, leading to a decline in muscle function and an increased risk of falls (21).

BMI and dominant handgrip strength are also considered in the predictive model. This is because in individuals with CKD, renal function decline leads to the excretion of albumin in urine, often accompanied by symptoms such as hypoalbuminemia and malnutrition. These conditions typically result in decreased muscle mass, making it challenging for the body to support its weight, thus increasing the risk of falls. BMI is the simplest and most readily measurable indicator for assessing nutritional status, while handgrip strength is the simplest and most readily measurable indicator for assessing sarcopenia (16, 25). Furthermore, several studies have also demonstrated the correlation between handgrip strength and falls, providing additional support to the conclusions (26–29).

It is noteworthy that cognitive function is often considered a predictive factor for fall risk (17, 30). However, in this study, the decline in cognitive function among individuals with CKD has not been included as a predictive factor in the model. However, in this study, after adjusting for multiple factors, cognitive function did not exhibit a significant association with falls. This may be due to the widespread occurrence of cognitive decline among individuals with CKD.

However, there are several limitations should be considered. First, the incidence of falls in our study may have been underestimated. Fall accidents were defined based on self-reported incidents of falling within the past 2 years, introducing the potential for memory bias. Fall incidents without injury might be overlooked or forgotten in such self-reports. Second, the nomogram was created using data from Chinese adults with CKD aged over 45 years. To validate the model’s suitability for other groups, including younger individuals or those from different ethnic backgrounds, additional data from external cohorts are required. Third, the nomogram did not encompass several potential predictors, including balance function and walking speed and so on, which could exert an influence on the occurrence of falls. However, our model is designed to be used in the community, focusing on markers that are both easily measurable and efficient for widespread application. The chosen predictors in our model, including fall down experience, BMI, mobility, dominant handgrip, and depression, align with the principle that applicable prediction models should leverage easily measurable characteristics. Fourth, this study outcomes are based on self-reported falls within 2 years, covering both injurious and non-injurious falls, but do not include fall frequency. Our main focus is on falls as isolated incidents. Although we acknowledge that non-injurious falls are also worthy of attention, considering the cost–benefit ratio, further research is needed to comprehensively evaluate a broader range of outcomes.

## 5 Conclusion

In this study, we developed and validated a predictive model for fall accidents among individuals with CKD in the community, presenting it in the form of a nomogram. Variables such as fall down experience, BMI, mobility, dominant handgrip, and depression were identified and included in the model. Internally validated, the model proved to be a useful tool for assessing the risk of fall incidents. The developed predictive model holds potential significance in screening CKD patients at a high risk of experiencing falls.

## Data availability statement

Publicly available datasets were analyzed in this study. This data can be found at: http://charls.pku.edu.cn/index.htm.

## Ethics statement

The studies involving humans were approved by Biomedical Ethics Review Committee of Peking University. The studies were conducted in accordance with the local legislation and institutional requirements. The participants provided their written informed consent to participate in this study.

## Author contributions

PL: Methodology, Writing – original draft. GL: Data curation, Writing – original draft. BW: Writing – original draft, Data curation. JZ: Formal analysis, Writing – original draft. MW: Writing – original draft, Formal analysis. FT: Writing – original draft, Conceptualization. LW: Data curation, Writing – original draft. YY: Data curation, Writing – original draft. LP: Funding acquisition, Writing – original draft. XL: Supervision, Writing – review & editing. LD: Writing – review & editing.
