# Supplementary material for: Development and validation of prediction model for fall accidents among chronic kidney disease in the community
Source: Front Public Health. 2024 May 30;12:1381754. doi: 10.3389/fpubh.2024.1381754 (PMC11171714; doi:10.3389/fpubh.2024.1381754)
Supplement: Supplementary file 1 [file Data_Sheet_1.docx]

**Supplemental Table 1** Baseline characteristics of the study population before interpolation.

| **Variables** | **Total (n = 911)** | **Non-fall**  **(n = 669)** | **Fall**  **(n = 242)** | ***P*** |
| --- | --- | --- | --- | --- |
| Gender, n (%) | 463 (50.9) | 351 (52.5) | 112 (46.3) | 0.095 |
| Age, Mean ± SD | 61.2 ± 9.0 | 60.8 ± 8.8 | 62.3 ± 9.5 | 0.032 |
| Married status, n (%) | 808 (88.8) | 606 (90.7) | 202 (83.5) | 0.002 |
| Disability, n (%) | 299 (32.8) | 208 (31.1) | 91 (37.6) | 0.064 |
| Alcohol Status, n (%) | 326 (35.8) | 237 (35.4) | 89 (36.8) | 0.707 |
| Smoke Status, n (%) | 341 (37.5) | 252 (37.7) | 89 (36.9) | 0.839 |
| BMI, n (%) |  |  |  | 0.429 |
| <18.5 | 54 (6.0) | 36 (5.5) | 18 (7.5) |  |
| 18.5-24.9 | 507 (56.6) | 366 (55.9) | 141 (58.8) |  |
| 25-29.9 | 283 (31.6) | 213 (32.5) | 70 (29.2) |  |
| ≥30 | 51 (5.7) | 40 (6.1) | 11 (4.6) |  |
| Mobility, n (%) | 56 (6.1) | 25 (3.7) | 31 (12.8) | < 0.001 |
| Toilet Seat Usage, n (%) | 28 (3.1) | 18 (2.7) | 10 (4.1) | 0.266 |
| Fall Down Experience, n (%) | 251 (27.6) | 130 (19.4) | 121 (50.2) | < 0.001 |
| Glaucoma, n (%) | 9 (1.0) | 5 (0.8) | 4 (1.7) | 0.257 |
| Pain, n (%) | 432 (47.9) | 290 (43.9) | 142 (59.2) | < 0.001 |
| Night Sleep Duration, Mean ± SD | 6.0 ± 2.0 | 6.1 ± 1.9 | 5.6 ± 2.2 | < 0.001 |
| ADL, n (%) |  |  |  | < 0.001 |
| Independent | 617 (67.9) | 484 (72.6) | 133 (55) |  |
| Dependent | 292 (32.1) | 183 (27.4) | 109 (45) |  |
| IADL, n (%) |  |  |  | < 0.001 |
| Independent | 600 (65.9) | 469 (70.1) | 131 (54.1) |  |
| Dependent | 311 (34.1) | 200 (29.9) | 111 (45.9) |  |
| CESD-10 Score, Mean ± SD | 10.8 ± 6.0 | 10.2 ± 5.8 | 12.6 ± 6.2 | < 0.001 |
| Cognitive Score, Mean ± SD | 10.7 ± 4.0 | 10.9 ± 3.9 | 9.9 ± 4.1 | 0.001 |
| Waist, Mean ± SD | 85.0 ± 15.2 | 85.4 ± 14.9 | 84.0 ± 16.0 | 0.221 |
| Dominant Handgrip (kg), Mean ± SD | 1 | 29.6 ± 9.5 | 26.6 ± 9.1 | < 0.001 |
| eGFR, Mean ± SD | 100.1 ± 28.0 | 99.8 ± 28.6 | 100.9 ± 26.6 | 0.606 |
| SBP, Mean ± SD | 127.5 ± 19.3 | 127.1 ± 18.7 | 128.7 ± 20.9 | 0.281 |
| DBP, Mean ± SD | 75.5 ± 11.6 | 75.5 ± 11.3 | 75.4 ± 12.3 | 0.892 |
| Pulse, Mean ± SD | 73.6 ± 10.5 | 73.7 ± 10.5 | 73.1 ± 10.5 | 0.431 |
| White Blood Cell, Mean ± SD | 6.1 ± 1.9 | 6.1 ± 1.9 | 5.9 ± 2.0 | 0.101 |
| Hemoglobin, Mean ± SD | 13.8 ± 2.1 | 13.8 ± 2.2 | 13.6 ± 1.8 | 0.067 |
| Platelets, Median (IQR) | 194.0 (154.0, 241.0) | 197.0 (155.8, 245.0) | 188.5 (147.5, 231.5) | 0.033 |
| TG, Median (IQR) | 110.6 (81.0, 165.9) | 111.5 (80.5, 166.4) | 104.9 (81.4, 162.6) | 0.34 |
| BUM, Median (IQR) | 15.1 (12.6, 18.2) | 15.1 (12.6, 18.5) | 15.1 (12.9, 17.9) | 0.571 |
| HDL, Median (IQR) | 50.6 (43.2, 58.7) | 49.8 (43.2, 57.9) | 52.1 (43.9, 60.2) | 0.063 |
| LDL, Median (IQR) | 101.2 (83.8, 119.1) | 100.4 (82.8, 118.2) | 102.9 (86.1, 121.3) | 0.139 |
| Glucose, Median (IQR) | 95.5 (88.3, 104.5) | 95.5 (88.3, 104.5) | 95.5 (88.3, 104.5) | 0.964 |
| UA, Median (IQR) | 4.9 (4.0, 5.9) | 4.9 (4.0, 5.9) | 4.8 (4.0, 5.8) | 0.733 |
| Cystatin C, Median (IQR) | 0.8 (0.7, 1.0) | 0.8 (0.7, 1.0) | 0.9 (0.8, 1.0) | 0.388 |
| C-reactive protein, Median (IQR) | 1.4 (0.8, 2.7) | 1.5 (0.8, 2.7) | 1.3 (0.7, 2.9) | 0.968 |
| HbA1c, Median (IQR) | 5.8 (5.5, 6.1) | 5.8 (5.5, 6.1) | 5.8 (5.5, 6.2) | 0.376 |
| Hypertension, n (%) | 361 (39.6) | 258 (38.6) | 103 (42.6) | 0.276 |
| Dyslipidemia, n (%) | 205 (22.5) | 149 (22.3) | 56 (23.1) | 0.782 |
| Diabetes, n (%) | 128 (14.1) | 91 (13.6) | 37 (15.3) | 0.518 |
| Cancer, n (%) | 26 (2.9) | 19 (2.8) | 7 (2.9) | 0.966 |
| Heart disease, n (%) | 279 (30.6) | 197 (29.4) | 82 (33.9) | 0.199 |
| Stroke, n (%) | 37 (4.1) | 24 (3.6) | 13 (5.4) | 0.228 |
| Depression Disease, n (%) | 26 (2.9) | 20 (3) | 6 (2.5) | 0.683 |
| Memory Related Disease, n (%) | 41 (4.5) | 23 (3.4) | 18 (7.4) | 0.01 |
| Arthritis, n (%) | 537 (58.9) | 372 (55.6) | 165 (68.2) | < 0.001 |
| Asthma, n (%) | 88 (9.7) | 58 (8.7) | 30 (12.4) | 0.093 |

*Note:* BMI, body mass index; ADL, activity of daily living; IADL, instrumental activities of daily living; CESD-10: Center for Epidemiologic Studies Depression Scale - 10 item version; SBP: systolic blood pressure; DBP: diastolic blood pressure; TG: triglycerides; BUM: blood urea nitrogen; HDL-C: high-density lipoprotein cholesterol; LDL-C: low-density lipoprotein cholesterol.; UA: uric acid.


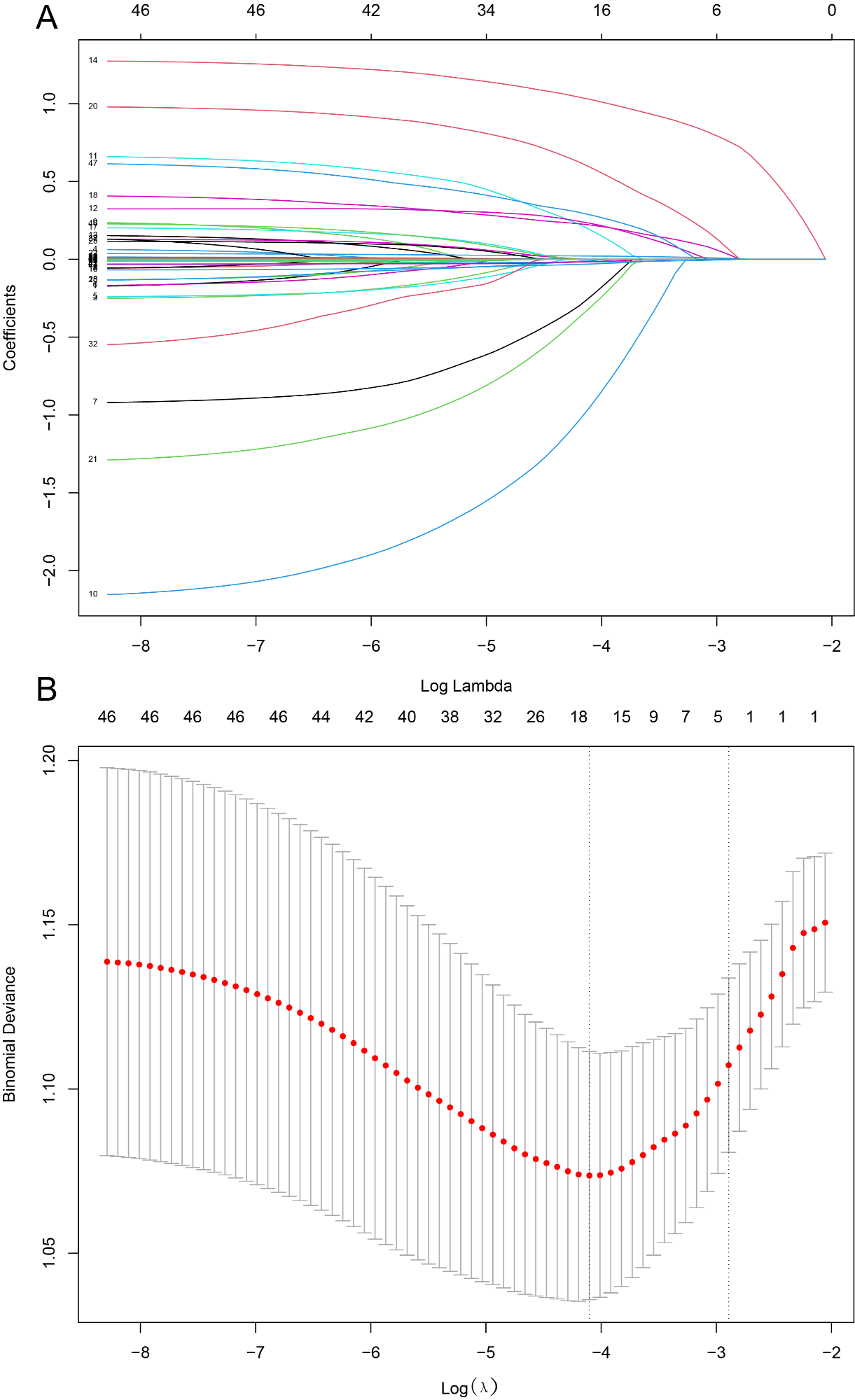


**Supplemental Figure 1** Predictive factors selection using the LASSO regression model.

**
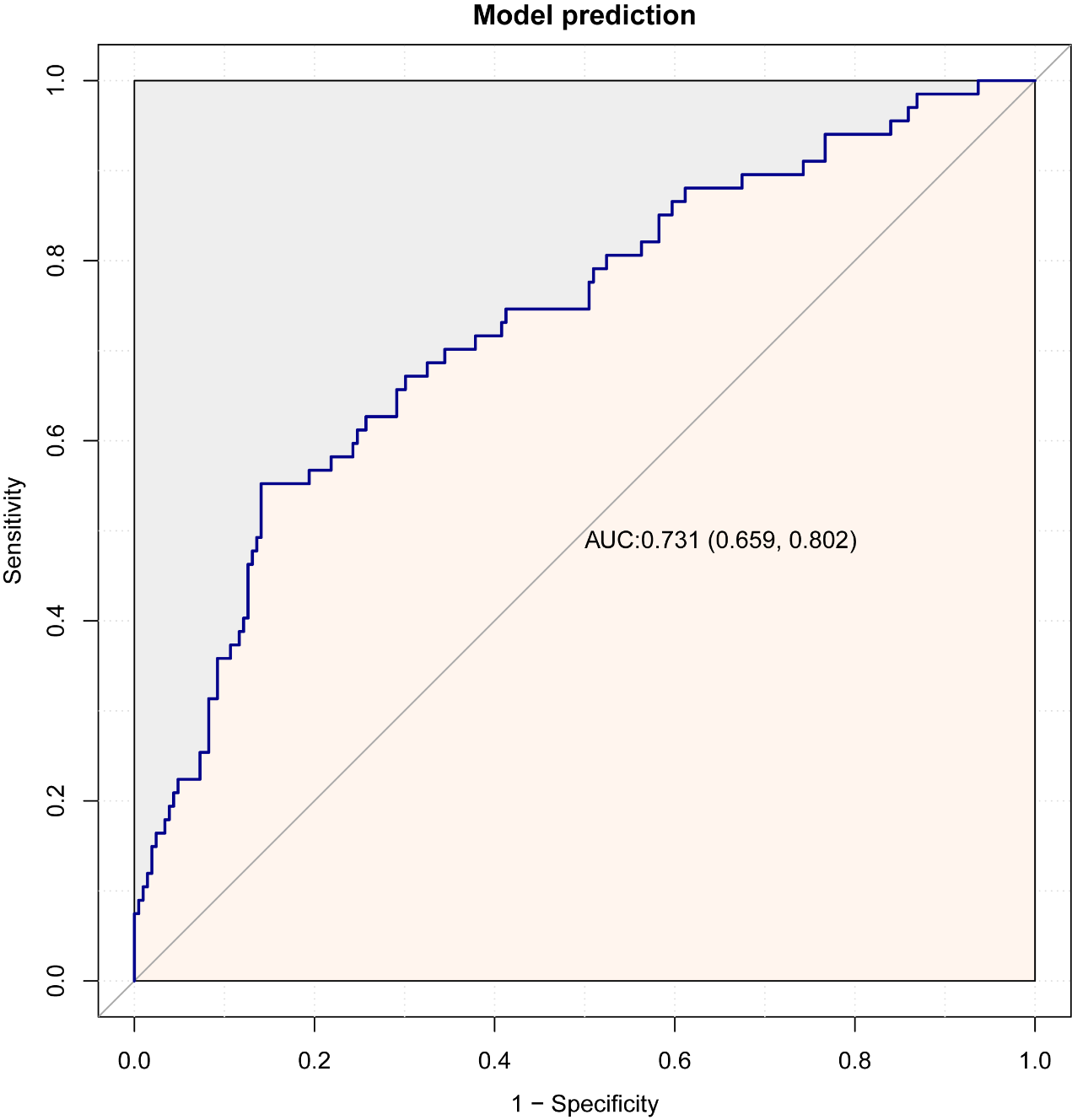
**

**Supplemental Figure 2** ROC curves plot for the validation dataset.

**
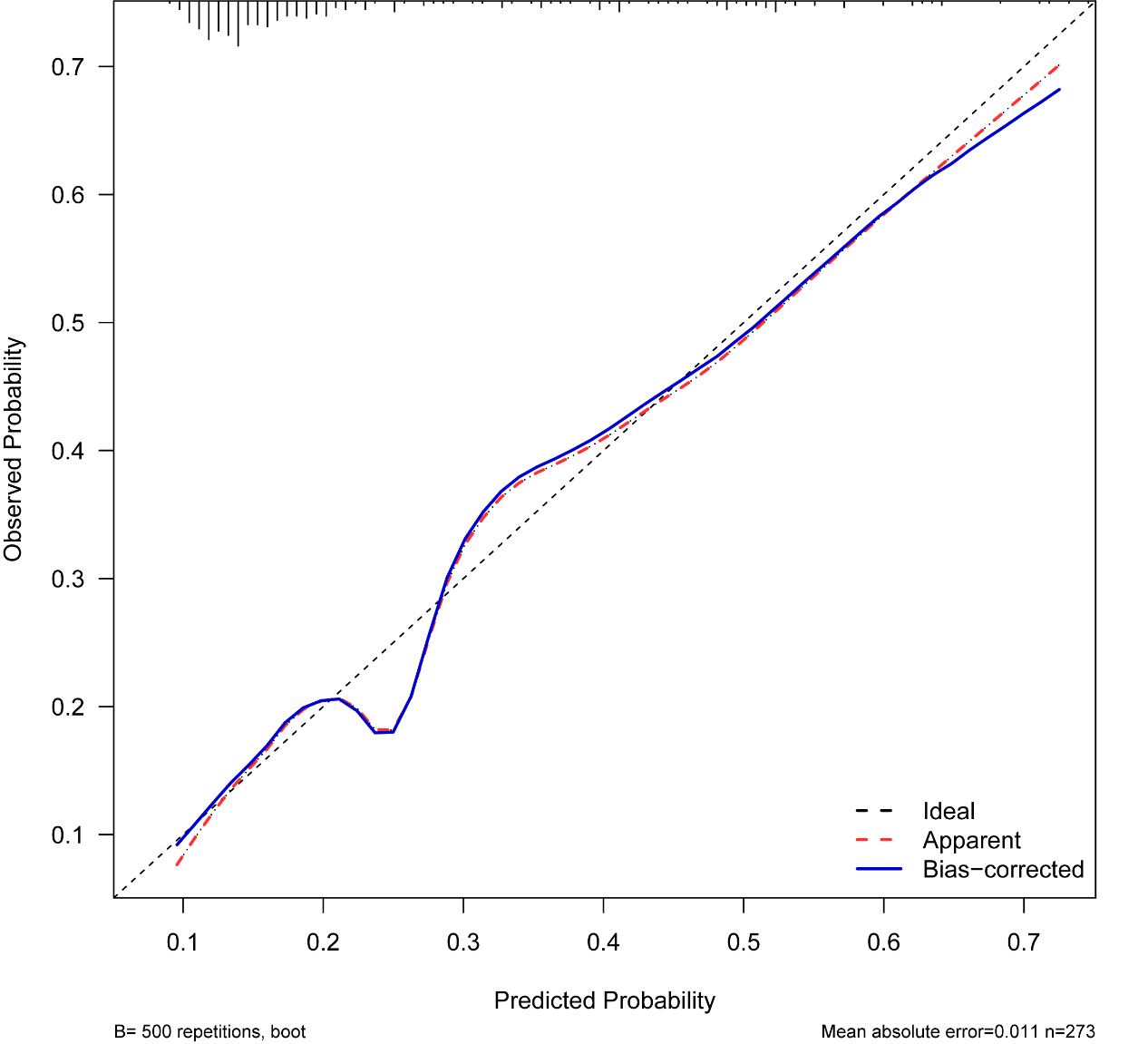
**

**Supplemental Figure 3** Calibration plot for the validation dataset.

**
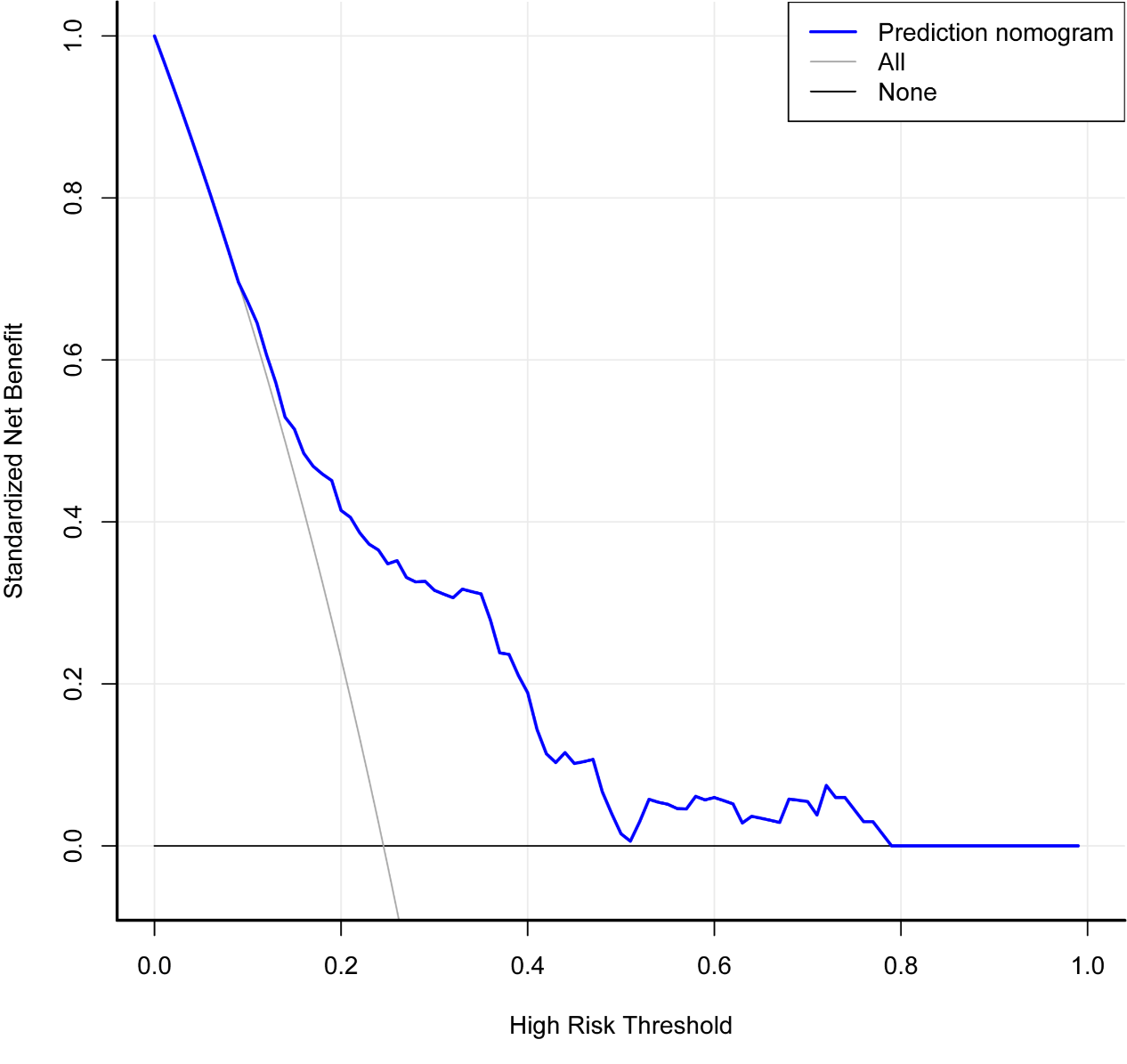
**

**Supplemental Figure 4** Decision curve plot for the validation dataset.
